# The Prognostic and Therapeutic Role of Histone Acetylation Modification in LIHC Development and Progression

**DOI:** 10.3390/medicina59091682

**Published:** 2023-09-18

**Authors:** Ji Gao, Sheng Han, Jian Gu, Chen Wu, Xiaoxin Mu

**Affiliations:** 1 Hepatobiliary Center, The First Affiliated Hospital of Nanjing Medical University, Nanjing 210029, Chinagujian@njmu.edu.cn (J.G.); 2 Key Laboratory of Liver Transplantation, Chinese Academy of Medical Sciences, Nanjing 210029, China; 3 NHC Key Laboratory of Living Donor Liver Transplantation, Nanjing 210029, China

**Keywords:** histone acetylation, LIHC, tumor microenvironment, immunotherapy, signature

## Abstract

*Background and Objectives:* The modification of histone acetylation plays a vital role in regulating tumor occurrence and development, but the interaction between histone acetylation modulator genes and the liver hepatocellular carcinoma (LIHC) microenvironment, as well as immunotherapy, has not been investigated. *Materials and Methods:* Analysis of all statistical data was carried out using R software (Version 4.2.0) and the online tool Sangerbox. Comprehensive bioinformatics analysis, including signature construction and validation, functional analyses, immune and genomic features analyses, and immunotherapy prediction analyses, were performed to explore the prognostic and therapeutic role of histone acetylation modulator genes in LIHC development and progression. *Results:* The LIHC cohort from The Cancer Genome Atlas (TCGA) database was selected as the training cohort; the GSE76427 cohort from the Gene Expression Omnibus (GEO) database and the LIRI-JP cohort from the International Cancer Genome Consortium (ICGC) database were selected as the validation cohorts. The histone acetylation modulator gene-based prognostic signature was constructed and validated successfully. Immune infiltration analysis showed that most immune cells and immune functions were enriched in patients with high histone acetylation risk scores (HARS). Additionally, high levels of checkpoint inhibitors (ICIs) and human leukocyte antigens (HLAs) were also observed in high HARS patients. Meanwhile, TIDE algorithm analysis was conducted to explore the relationship between HARS and immunotherapy response, and submap algorithm analysis was used for the verification of the results, from which we found that high HAPS patients were more likely to respond to immunotherapy. *Conclusions:* Our findings revealed that the histone acetylation modulator genes, particularly for KAT21, SIRT6, and HAT1, may have the potential to function as a new prognostic marker and therapeutic target for LIHC.

## 1. Introduction

Liver cancer ranks seventh in the list of most commonly occurring cancers and has the second-highest death rate [1]. Liver cancer is on the rise worldwide, and it is estimated that 1 million people will be impacted by liver cancer by 2025. The most common type of liver cancer is liver hepatocellular carcinoma (LIHC), which accounts for 80–90% of primary liver cancer cases, and intrahepatic cholangiocarcinoma (ICC) accounts for approximately 10% [2]. A majority of LIHC patients experience recurrence after regional resections, and only a few patients qualify for transplants due to undiagnosed disease or being found too late to be cured. The treatment of LIHC with systemic molecular therapies, regional resections, and liver transplants remains unsatisfactory and limited [3,4,5]. A variety of immunotherapies, such as immune checkpoint inhibitors, tyrosine kinase inhibitors, and monoclonal antibodies, are revolutionizing the management of LIHC today. Despite the encouraging results of studies showing an improvement in overall survival rates and quality of life for LIHC patients, some still cannot benefit from immunotherapy due to tumor heterogeneity [5]. Changing treatment strategies at different stages of LIHC is something we look forward to.

Mutations in genes and epigenetic changes in gene expression contribute greatly to liver cancer carcinogenesis [6]. Instead of directly affecting the DNA coding sequence, epigenetics could influence gene expression through heritable chromatin modifications [7]. A variety of epigenetic mechanisms can regulate gene expression, including acetylation, methylation, phosphorylation, ubiquitylation, and sumoylation of histone proteins. Histone acetylation, promoting greater accessibility of transcription factors and RNA polymerase II to help genes initiate transcription, is a reversible process that adds acetyl groups from acetyl CoA to the Ɛ-amino group on lysine residues of histone tails [8]. Proliferation, metastasis, and invasion of cancer cells are associated with histone acetylation, which is regulated by histone acetyltransferases (HATs) and histone deacetylases (HDACs) [9,10]. In cancer, HDAC overexpression leads to the silencing of the tumor suppressor genes (TSG) and abnormal transcription and may be targeted in treatment [11]. A growing body of evidence suggests that epigenetic marks can help identify cancer biomarkers for the early diagnosis and treatment of liver cancer.

In recent years, there a well-established relationship has been identified between epigenetic modifications, including N^7^-methylguanosine (m7G), N^6^-methyladenosine (m6A), N^1^-methyladenosine (m1A), and 5-methylcytosine (m5C), and tumor development and progression. However, the modification of histone acetylation in tumor is rarely investigated, especially in LIHC, and whether the level of histone acetylation is involved in the regulation of TME and immunotherapy in LIHC remains obscure. In this study, we performed comprehensive bioinformatic analyses, including signature construction and validation, functional analyses, immune and genomic features analyses, and immunotherapy prediction analyses to explore the potential prognostic and therapeutic markers for LIHC based on histone acetylation modulator genes.

Histone acetylation modification plays a vital role in the development and progression of LIHC. In this study, we conducted a series of bioinformatics analyses to explore the potential prognostic and therapeutic role of histone acetylation modulator genes in LIHC.

## 2. Materials and Methods

### 2.1. Describe the Expression Landscape of Histone Acetylation Modulator Genes

RNA sequencing data and relevant clinical information of all LIHC samples were downloaded from the TCGA database for the training cohort. Two independent LIHC cohorts, including the GSE76427 cohort from the GEO database and the LIRI-JP cohort from the ICGC database, were included as validation cohorts. Based on previous literature, 73 histone acetylation modulator genes in total were captured (Supplementary Table S1) [12]. The first step was to observe the expression of all histone acetylation regulatory genes across all samples. Following that, univariate regression analysis and differential expression analysis were performed on these histone acetylation modulator genes. We then quantified the score of histone acetylation for each sample based on the ssGSEA algorithm and mapped the distribution of the score with different clinical features. In the final step, we analyzed the difference in histone acetylation score between LIHC and normal samples.

### 2.2. Development of a Prognostic Signature Based on Histone Acetylation Modulator Genes

As well as univariate regression analysis, lasso regression analysis was performed on these genes, and the results were selected simultaneously for the multivariate regression analysis. As a result, three histone acetylation modulator genes, including KAT2A, SIRT6, and HAT1, were screened for the construction of the prognostic signature, and a formula for calculating histone acetylation risk score (HARS) was developed. Then, using the “maxstat” package algorithm based on R software, an optimal cut-off of HARS was determined, based on which LIHC patients were divided into high HARS and low HARS groups. Due to the importance of these three genes in the signature, we tested their differential expression in paired samples, and we also conducted Kaplan–Meier (KM) survival analyses of these signature genes separately.

### 2.3. Verification of the Histone Acetylation Modulator Gene-Based Signature

To verify the reliability of our signature, ROC analysis was carried out in three cohorts, respectively, to verify the signature performance, and the relationship between the level of HARS and the survival status of samples was plotted. Further analysis was conducted with samples of different clinical traits in order to determine whether the expression of signature genes and HARS was differentially expressed. In order to improve the effectiveness of HARS to clinical application, five different clinical features, including survival time and survival status, were combined to develop a nomogram, and we evaluated the prognostic significance of these features in LIHC samples lastly.

### 2.4. Functional Analyses and Genome Analyses

To explore differences and connections among two groups from the potential molecular mechanisms, gene ontology (GO) and Kyoto Encyclopedia of Genes and Genomes (KEGG) analyses were performed based on the top 200 differentially expressed genes. Following that, the top 20 genes with the highest mutation frequency between the two HARS groups were presented with a waterfall plot pattern. For the assessment of the copy number variations (CNV) differences, the amplification and deletion frequency between the two groups were compared between two groups. Furthermore, we also used differential expression analysis to identify the burden CNV differences at both focal and arm levels between two HARS groups.

### 2.5. Analysis of Immune Infiltration and Prediction of Immunotherapy

In the first step, a total of 70 immune signatures were quantified for each patient using the xCell algorithm. Additionally, correlation analyses were conducted using the ESTIMATE algorithm to assess the relationship between HARS and ESTIMATE, stromal, and immune scores. Since the signature genes are important, we also analyzed the correlation between the expression of three signature genes and these immune signatures. Meanwhile, we also compared the expression of different common immune checkpoints and human leukocyte antigens between the two groups to determine if cancer treatment relies heavily on them. In the next step, predictions of immunotherapy response among TCGA LIHC samples were made based on the TIDE algorithm, and verification for the credibility of immunotherapy prediction results was achieved with the submap algorithm [13,14].

## 3. Results

### 3.1. The Expression Landscape of Histone Acetylation Modulator Genes in Samples

**Figure 1:**
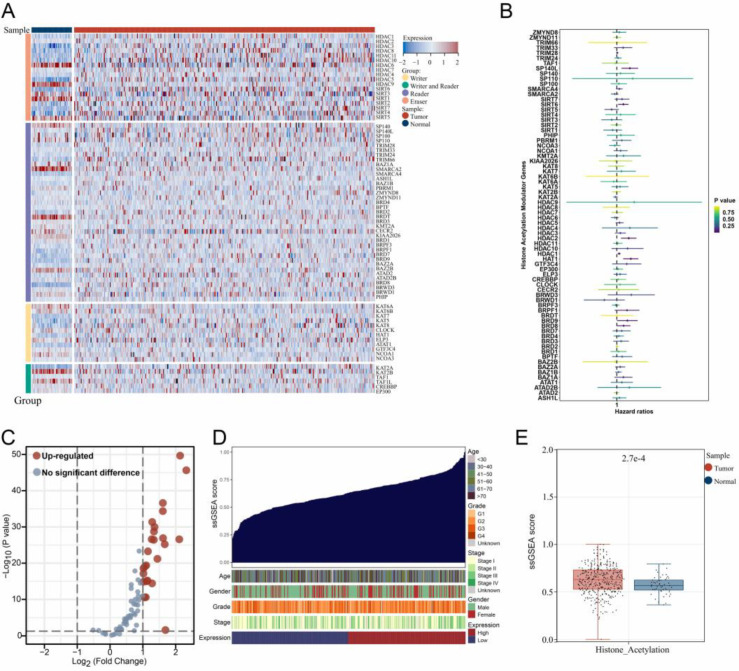
Expression patterns of histone acetylation modulator genes in samples. Notes: (**A**) Heatmap of the expression of 73 histone acetylation modulator genes in both LIHC samples compared to normal. (**B**) Univariate Cox regression analysis of 73 histone acetylation modulator genes. (**C**) The volcano plot of differential 73 histone acetylation modulator genes expression analysis. (**D**) Distribution of the histone acetylation scores and the associations with different clinical features. (**E**) Differential expression analysis of histone acetylation scores between LIHC and normal samples.

After the acquisition of all histone acetylation modulator genes, a heatmap was drawn separately for different groups of these genes in both LIHC samples compared to normal (Figure 1A). Following that, the univariate Cox regression analysis selected nine genes affecting prognosis (Figure 1B; Supplementary Table S2), and differential expression analysis revealed that most histone acetylation modulator genes were upregulated in LIHC samples (Figure 1C). Then, the distribution of the histone acetylation scores in LIHC samples was ordered from low to high to survey the associations between the scores and different clinicopathological features (Figure 1D). Finally, the results of differential expression analysis showed that LIHC samples had a higher level of histone acetylation score (Figure 1E).

### 3.2. Signature Construction Based on Histone Acetylation Modulator Genes

**Figure 2:**
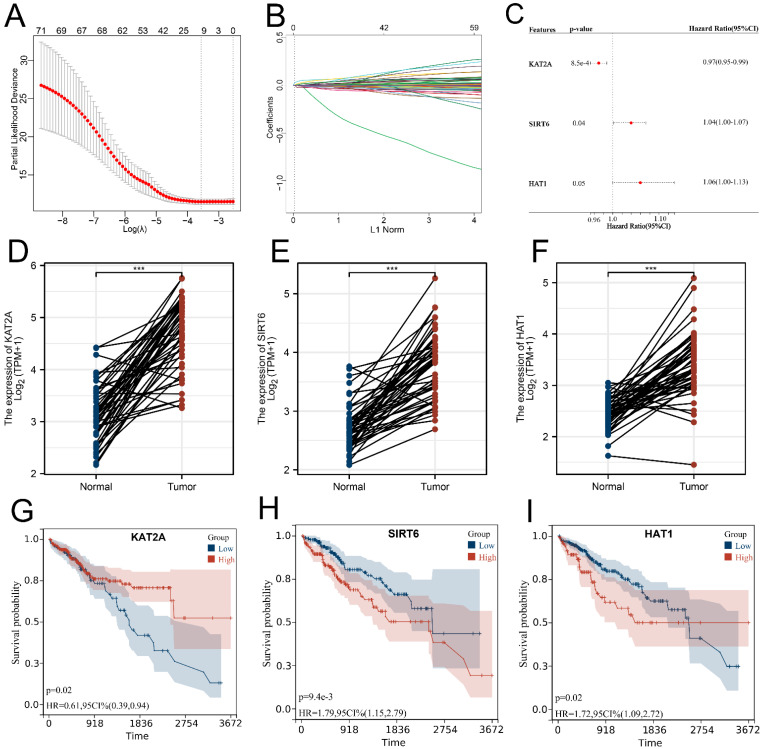
Construction of a prognostic signature and calculation of HARS. Notes: (**A**,**B**) Lasso regression analysis results of 73 histone acetylation modulator genes. (**C**) Multivariate Cox regression analysis identified three genes for the signature construction. (**D**–**F**) Differential expression analysis of three signature genes between LIHC and normal samples. (**G**–**I**) Kaplan–Meier (KM) survival curves analysis of three signature genes. *** indicates *p* < 0.001.

Given more genes need to be obtained for the signature construction in order to increase the predictive accuracy, we opted to additionally perform lasso regression analysis and 11 genes were selected for further analysis (Figure 2A,B). After deduplication, a total of 15 genes were included to conduct multivariate Cox regression analysis, and a prognostic signature composed of three genes including KAT2A, SIRT6, and HAT1 was successfully constructed with a HARS formula: HARS = the expression of KAT2A^×^ − 0.0309014374534181 + the expression of SIRT6^×^ 0.035265783612255 + the expression of HAT1^×^ 0.0628702601593971 (Figure 2C; Supplementary Table S3). In LIHC samples, each signature gene was overexpressed (Figure 2D–F). Furthermore, Figure 2G–I shows the general survival probability of patients using KM curves analysis based on these signature genes, and we found that patients with higher levels of KAT2A, lower levels of SIRT6, or lower levels of HAT1 had better survival.

### 3.3. Verification and Evaluation of the Signature

**Figure 3:**
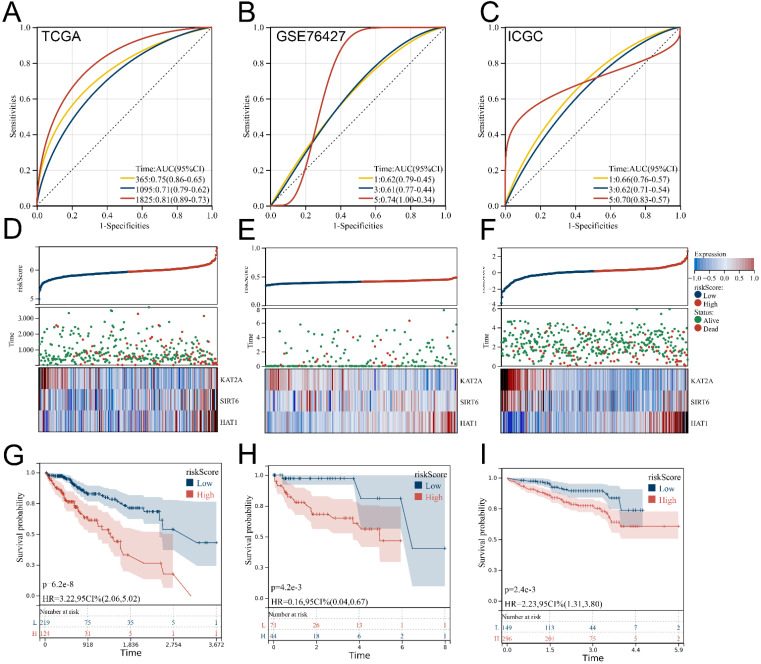
External validation of the signature based on two independent LIHC cohorts. Notes: (**A**–**C**) The time-dependent ROC analysis showed excellent predictive ability of HARS in both testing cohorts and two independent external cohorts. (**D**–**F**) Relationship analysis was examined to see how the level of HARS correlated with the OS time, survival status, and the expression of the signature genes. (**G**–**I**) KM survival curves analysis based on HARS in both the testing cohort and two independent external cohorts.

**Figure 4:**
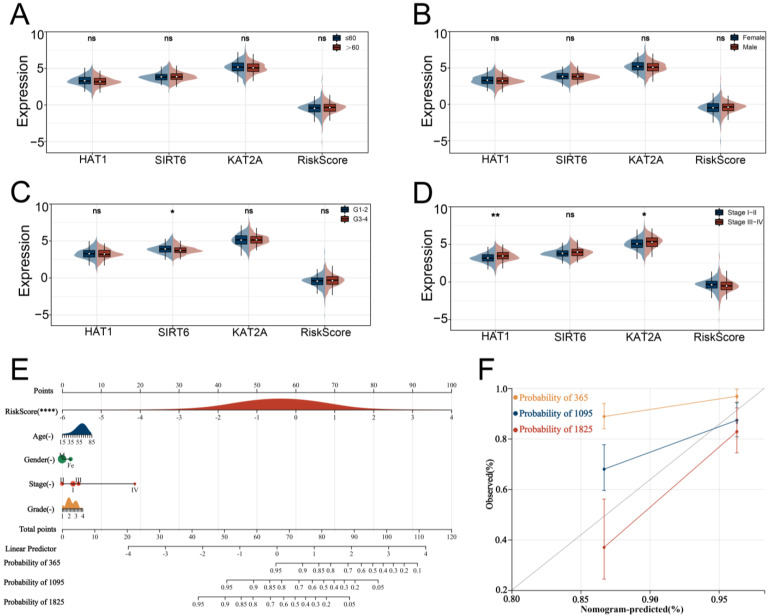
Clinical association study and development of a nomogram. Notes: (**A**–**D**) Association Analysis between HRAS and different clinical features revealed that patients with stage III-IV had a higher level of HAT1, while other comparisons are not statistically significant. (**E**) Development of a nomogram based on HARS and different clinical features. (**F**) The nomogram showed good predictive ability of 1–3–5 years with a C-index of 0.723678870038152. ns indicates *p* > 0.05, * indicates *p* < 0.05, ** indicates *p* < 0.01, **** indicates *p* < 0.0001.

In addition to the LIHC samples included from the TCGA database as the testing cohort, we obtained the GSE76427 cohort from the GEO database and the LIRI-JP cohort from the ICGC database as external validation cohorts. To enhance predictive benefits, the optimal cut-off of −0.126210861818856 for HARS was identified based on the “maxstat” package algorithm. Time-dependent ROC curve analyses showed the overall accuracy was excellent among all three cohorts, with a satisfactory predicted efficacy on patient prognosis in the TCGA group (Figure 3A; 1-year AUC = 0.75, 3-year AUC = 0.71, 5-year AUC = 0.81). Additionally, our prognosis model showed an effectively predicted efficacy in the GEO group (Figure 3B; 1-year AUC = 0.62, 3-year AUC = 0.61, 5-year AUC = 0.74) and ICGC group (Figure 3C; 1-year AUC = 0.66, 3-year AUC = 0.62, 5-year AUC = 0.70). From Figure 3D–F, we can see that when the level of HARS was higher, the risk of mortality for patients was higher. Additionally, KM survival curve analyses of the OS-based HARS between the two groups were performed, and the concordance was observed to a high degree (Figure 3G–I). In clinical association analyses between HARS and different clinicopathological features, it should be noted that both HAT1 and KAT2A are highly expressed at advanced TNM stages (Figure 4A–D). Considering HARS needs to be applied to clinical settings in order to be more effective, we developed a nomogram consisting of HARS and four clinical features with a C-index of 0.723678870038152 (Figure 4E,F).

### 3.4. GO and KEGG Analyses and Genomic Features Analysis

**Figure 5:**
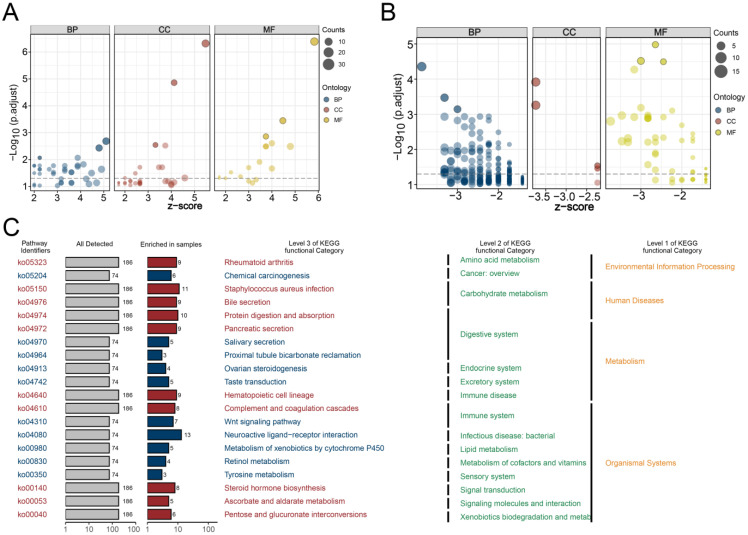
Pathway enrichment analysis, including GO and KEGG. Notes: Based on GO analysis, differences in biological pathways between high HARS patients (**A**) and low HARS (**B**) patients were explored. (**C**) The results of the KEGG analysis showed different functional categories enriched in two HARS groups, and all the terms were classified according to the annotation information of the three levels on the KEGG website.

**Figure 6:**
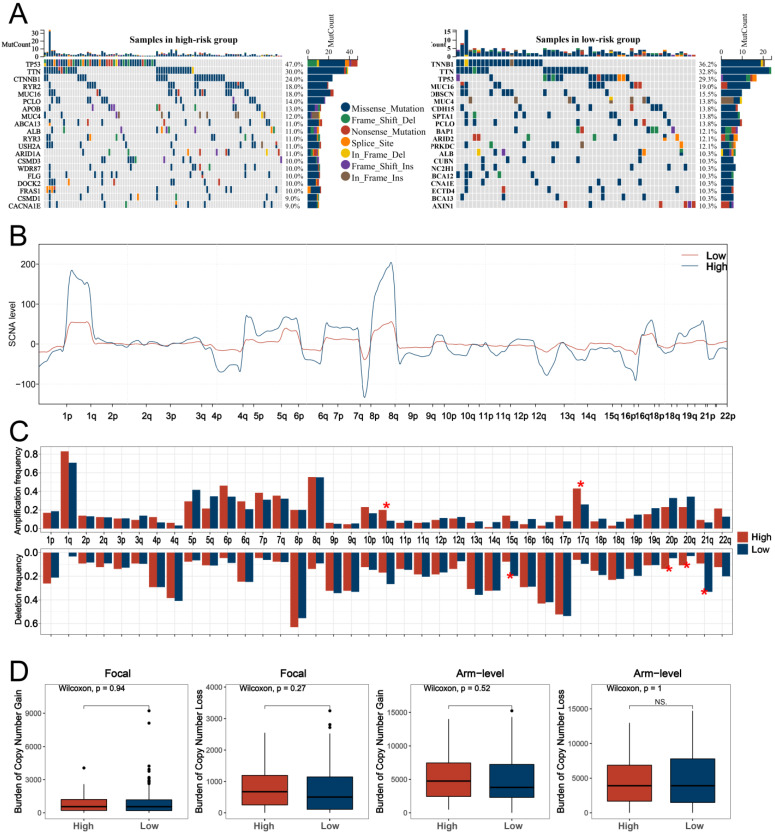
Genomic characterization analysis between two HARS groups. Notes: (**A**) Two waterfall diagrams were used to identify differences in mutations between the high HARS group and the low HARS group for 20 genes. (**B**) Detailed information on arm-level SCNA between the two HARS groups. (**C**) The differences in amplification and deletion frequency between the two groups. (**D**) Differential expression analysis of the burden CNV differences at both focal and arm levels between the two HARS groups. ns indicates *p* > 0.05, * indicates *p* < 0.05.

Based on the top 200 differentially expressed genes selected from two HARS groups, GO analysis was performed, and the top three pathways in biological process (BP), molecular function (MF), and cellular component (CC) were shown in both the high HARS group (Figure 5A) and low HARS group (Figure 5B). Both GO analysis and KEGG analysis were conducted, and the results showed different functional categories were enriched in the two HARS groups. In addition, taking into consideration that some terms had similar functions, all the terms were classified according to the annotation information of the three levels on the KEGG website (Figure 5C). Moreover, the waterfall diagram revealed differences between the two groups in mutations of the top 20 genes (Figure 6A). For CNV analysis, our study presented the gene-level status of SCNA in LIHC samples between the two HARS groups (Figure 6B), and we found that high HARS patients had higher 10q and 17q amplification frequency and 15q, 20p, 20q, and 21q deletion frequency (Figure 6C). Then, we also performed differential expression analysis of the burden of CNV at both focal and arm levels between two HARS groups that showed no discernible differences (Figure 6D).

### 3.5. Multiple Immune Analyses Revealed High HARS Patients More Sensitive to Immunotherapy

**Figure 7:**
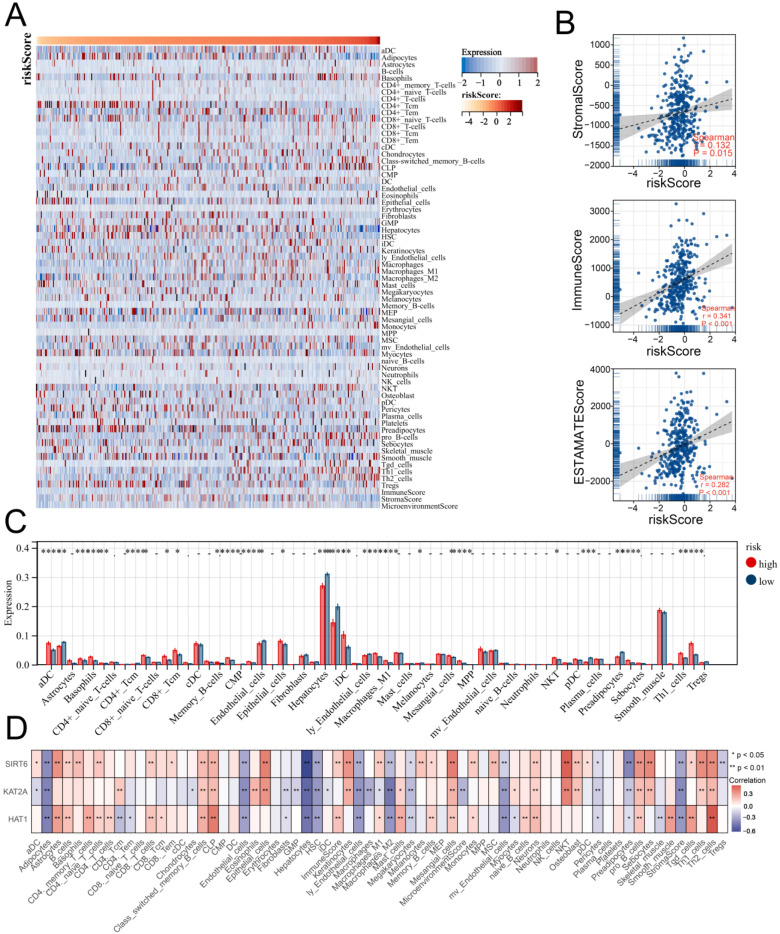
The infiltration of immune cells and immune functions between the two HARS groups. Notes: (**A**) Correlations between the level of 70 immune terms and HARS using the xCell algorithm. (**B**) Patients with high HARS had a high level of ESTIMATE score, stromal score, and immune score based on the ESTIMATE algorithm. (**C**) Differential expression analysis of the level of 70 immune terms between the two HARS groups. (**D**) Correlations between the expression of signature genes and the level of 70 immune terms. * indicates *p* < 0.05.

**Figure 8:**
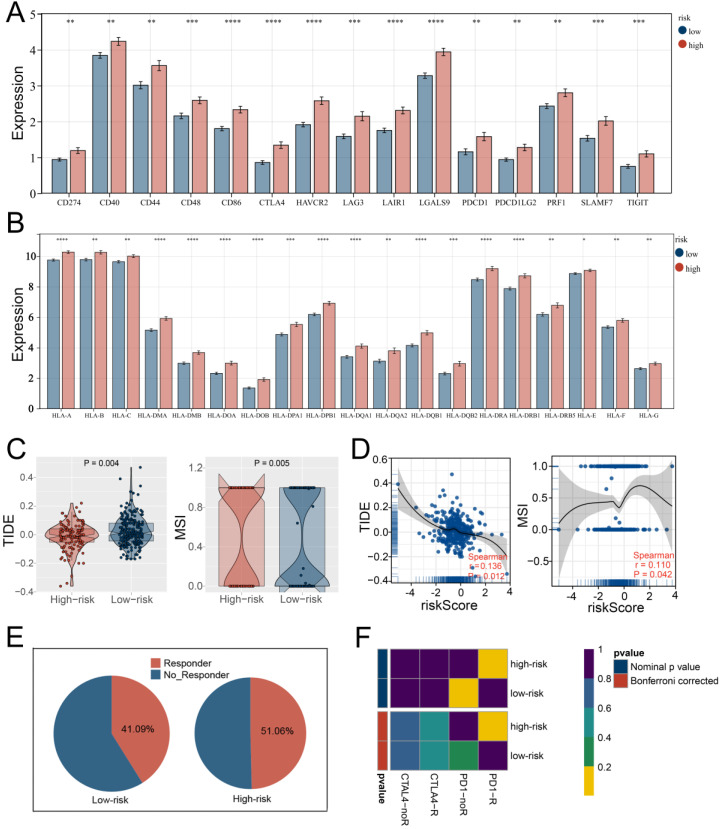
Immune term-based model associated with immunotherapy response. Notes: (**A**,**B**) The expression differences of immune checkpoints and HLAs between two HARS groups. (**C**) Patients with high HARS had a lower TIDE score and a higher MSI score. (**D**) HARS was negatively correlated with TIDE and positively correlated with MSI. (**E**) Patients in the high HARS group have a higher percentage of responders. (**F**) Submap analysis showed patients with high HARS were more sensitive to anti-PD-1 immunotherapy. * indicates *p* < 0.05, ** indicates *p* < 0.01, *** indicates *p* < 0.001, **** indicates *p* < 0.0001.

Based on the xCell algorithm, the level of 70 immune signatures of each LIHC sample were quantified and are shown in Figure 7A, indicating the correlations between HARS. In addition, we found that HARS was positively and strongly correlated with the ESTIMATE score and immune score using the ESTIMATE algorithm with *p* < 0.001 (Figure 7B). Meanwhile, high levels of most immune signatures were observed in high HARS patients (Figure 7C; Supplementary Table S4), and positive correlations between the expression of signature genes and the level of most immune signatures were also found (Figure 7D). In light of the importance of immunotherapy with immune checkpoint inhibitors (ICIs) and human leukocyte antigens (HLAs), the expression of common immune checkpoints and HLAs was further investigated in two HARS groups, and the results showed high HARS patients had a higher level of immune checkpoints and HLAs (Figure 8A,B). To determine whether high HARS patients could be more sensitive to cancer immunotherapy, the TIDE algorithm was used to predict the immunotherapy response, from which we observed that high HARS patients had a lower TIDE score and a higher MSI score, indicating that patients with high HARS were more likely to respond to immunotherapy (Figure 8C,D). Alongside this, a higher percentage of responders was found in the high HARS group (Figure 8E). Finally, to further validate our findings, submap analysis was performed, and the results revealed that patients with high HARS were more sensitive to anti-PD-1 immunotherapy, which was consistent with the TIDE analysis (Figure 8F).

## 4. Discussion

The majority of nontumor cells in the LIHC microenvironment are tumor-infiltrating immune cells. Tumors with immunoinvasive characteristics survived longer and had lower recurrence rates after resection and transplantation [15]. LIHC with immune activation makes up about 30% of early cases, while those without immune invasion make up 25% [16]. Identifying and eliminating cancer cells in the tumor microenvironment is the main function of the immune system, but cancer cells can evade killing by overexpressing inhibitory ligands. The interactions between cancer cells and the immune system determine whether cancer growth is promoted or inhibited. In the fight against cancer, immunotherapy, including the blockade of immune checkpoints, appears to be a robust therapeutic approach [17]. Patients with LIHC still need to be observed for sensitivity to immune checkpoint blockades. According to studies, epigenic modifying agents like histone deacetylase inhibitors can enhance antitumor activity by increasing immune checkpoint expression during immunotherapy [18].

Histone acetylation affects chromatin compaction by adding acetyl groups to lysine residues at the tails, neutralizing the basic charge at unmodified lysine residues and at last, weakening the electrostatic interaction between negatively charged DNA and histones [19]. Histone acetylation is considered to accelerate gene transcription, especially acetylation of H4 at lysine (K)16, which has a considerable impact on cancer characteristics in various cancers [20,21]. As a result of transcription factors recruiting HDACs to deactivate promoter regions, onco-suppressor genes such as p21 can be inhibited, which is crucial for tumor metastasis or invasion [22]. Currently, pharmacology can reverse histone acetylation by inhibiting HDACs or HATs, which might be useful in developing anti-cancer treatment strategies. Identifying novel biomarkers in histone acetylation and their interaction with transcription factors that cause abnormal alterations associated with liver cancer will thus be helpful in finding viable strategies for diagnosis and treatment of LIHC [23].

In this study, three histone acetylation modulator genes, including KAT2A, SIRT6, and HAT1, were identified to develop the prognostic signature for LIHC patients. Among these genes, KAT2A was recently identified as a vulnerability in some forms of acute myeloid leukemia [24]. SIRT6 is implicated in cancer progression and onset from dual-sided effects since both blocking and inducing apoptosis result in opposite outcomes [25]. In addition, HAT1 expression was downregulated in LC cells, which contributed to lung cancer (LC) pathogenesis [26]. Furthermore, it is worth mentioning that HAT1 and KAT2A were highly expressed in advanced TNM stages, suggesting the expression of these signature genes may be associated with advanced-stage and high-grade carcinomas of LIHC. Not only that, this study demonstrates a novel nomogram which is more quantitative and intuitive, making it more convenient for clinicians to use. Two HARS groups showed no discernible differences in amplification/deletion frequency or the burden of CNV. However, in GO and KEGG analyses, we found that staphylococcus aureus infection, bile secretion, protein digestion and absorption, pancreatic secretion, steroid hormone biosynthesis, ascorbate and aldarate metabolism, and pentose and glucuronate interconversions pathways were enriched in patients with high HARS, indicating that these pathways could affect patient outcomes and be implicated in cancer pathways.

As is well-known, the field of tumor immunology has been revitalized by immunotherapy. It is possible to achieve durable clinical responses with immunotherapy, including ICIs; however, the efficacy of these treatments may differ from one case to another [27]. Furthermore, immunotherapy is only effective in certain subsets of cancer patients, and there is no clarity as to exactly who the target population is. A complex algorithm called TIDE was designed to predict patient immunotherapy responses and model immune evasion. Therefore, we used the TIDE algorithm to distinguish LIHC patients who are more sensitive to immunology, and we found that patients with high HARS had a lower TIDE score and a higher MSI score, suggesting that these patients may be more sensitive to immunology. A subclass mapping algorithm was also applied to evaluate similarities in responding to immunotherapies. To make the results more persuasive, a submap algorithm was performed to validate this finding, from which we were pleased to find that patients with high HARS were more sensitive to anti-PD-1 immunotherapy. We demonstrate the potential of our novel immune classification to provide new evidence for tumor-targeted treatments as a result of confirming our findings using the TIDE algorithm and subclass mapping methods. All these results revealed that HARS could serve as a prognostic and therapeutic biomarker for LIHC patients.

Though the results of our study appear to be promising, some inadequacies still need to be addressed. Firstly, there are many methods for immune cell infiltration; in addition to the xCell algorithm, more algorithms must be analyzed. Ideally, experimental verification is required to verify our findings in the future.

## 5. Conclusions

In conclusion, this study developed a prognostic signature based on histone acetylation modulator genes and investigated the clinical relevance and biological functions of patients between the two HARS groups. In further analysis, TIDE and submap algorithms were performed, and the results indicate that patients with high HARS were more likely to respond to immunotherapy.

## Data Availability

All the data used in our study are included in the article/Supplementary Material, and further inquiries can be directed to the corresponding author.

## Supplementary Materials

The following supporting information can be downloaded at: https://www.mdpi.com/article/10.3390/medicina59091682/s1, Table S1: List of the HAMPs in the human genome; Table S2: Results of univariate regression analysis; Table S3: Results of multivariate regression analysis; Table S4: Results of differential expression in immunoinfiltration analysis.
